# The early after discharge cardiac CT for low-risk chest pain study: the ED-CT study

**DOI:** 10.1093/bjr/tqae119

**Published:** 2024-06-18

**Authors:** Michael Cronin, Aisling Gill, Eve Blake, Niamh Dunne, Niall Sheehy, Geraldine McMahon, Ross Murphy, Caroline Daly

**Affiliations:** Department of Cardiology, St James Hospital, Dublin D08 NHY1, Republic of Ireland; Department of Cardiology, St James Hospital, Dublin D08 NHY1, Republic of Ireland; Department of Cardiology, St James Hospital, Dublin D08 NHY1, Republic of Ireland; Department of Cardiology, St James Hospital, Dublin D08 NHY1, Republic of Ireland; Department of Radiology, St James Hospital, Dublin D08 NHY1, Republic of Ireland; Department of Emergency Medicine, St James Hospital, Dublin D08 NHY1, Republic of Ireland; Department of Cardiology, St James Hospital, Dublin D08 NHY1, Republic of Ireland; Department of Cardiology, St James Hospital, Dublin D08 NHY1, Republic of Ireland

**Keywords:** pathway, outpatient, cardiac CT, low risk

## Abstract

**Objectives:**

An accelerated diagnostic pathway is created to aid the management of low-risk patients presenting to the emergency room with chest pain. Records are taken of patient outcomes and factors influencing physician decision-making between inpatient invasive angiography versus early outpatient cardiac CT angiography.

**Methods:**

A cohort study at 30 days post discharge is undertaken over 1 year. Differences are observed between a population of patients who underwent early outpatient CT and a population of ambulatory haemodynamically stable patients who underwent inpatient fluoroscopic angiography.

**Results:**

Totally, 369 patients underwent CT (*F* = 46%) and 37 underwent angiography (*F* = 30%). Median outpatient CT was at 14 days. At 30 days, 0 patients suffered mortality or myocardial infarction. Eleven percent were recommended for invasive angiography. Two percent of CT patients underwent coronary revascularization. Median calcium score was 0. Twenty percent of the CT population were commenced on high-potency statin or had their pre-existing statin dose intensified. Calcium score affected a composition of statin commencement, angiography, and revascularization (OR 59, *P* < .001). Age, troponin, vascular disease, and previous coronary revascularization appeared to influence choice between coronary computed tomography angiography (CCTA) and invasive angiography.

**Conclusion:**

An accelerated diagnostic pathway for outpatient cardiac CT for chest pain resulted in no mortality or myocardial infarction, with a low level of downstream testing and coronary revascularization.

**Advances in knowledge:**

At a median time to CCTA of 14 days post discharge from the emergency department, there is no effect on patient major adverse cardiac events.

## Introduction

Coronary computed tomography angiography (CCTA) is a recognized method of improvement in the diagnosis of angina pectoris and in the treatment of non-obstructive coronary artery disease.^1^ It is safe and cost effective in use in emergency departments (EDs).^2^^,^^3^ To optimize discharge time and appropriately stratify patient risk for subsequent cardiac events, a chest pain assessment accelerated diagnostic pathway was developed in conjunction between the cardiology department and ED in our facility since November 2021. The aim is to standardize care, encourage effective decision-making, and promote cost effectiveness in the management of low-risk cardiac patients presenting to the ED.

Given the clinical assessment of patients would be by independent nurse practitioners under the jurisdiction of the ED, the protocol included the “History, Electrocardiogram, Age, Risk Factors, Troponin levels” score (ie, HEART). This score has been included in societal guidelines for use in this population.^4^ This was incorporated with an evidence-based approach of a 0 and 3 h high-sensitivity cardiac troponin (hs-cTn).^5^^,^^6^

Within the protocol, the independent nurse practitioners identify a patient with chest pain and perform a structured cardiac assessment. This includes an electrocardiogram (ECG) within 10 min of arrival^7^ with clinical review by the independent nurse practitioner or ED doctor, and a standard serum profile for laboratory assessment (full blood count, renal and liver profile, hs-cTn, coagulation profile, and venous blood gas). If ST segment elevation is noted, then this will be dealt immediately via an urgent referral to in-house cardiology services. If this is not the case, then the nurse practitioners will record a clinical history (including coronary artery risk factors) and comprehensive physical assessment. As appropriate, adjunctive tests can be ordered and performed (eg, chest X-ray, transthoracic echocardiogram, interval ECG).

**Figure 1.**
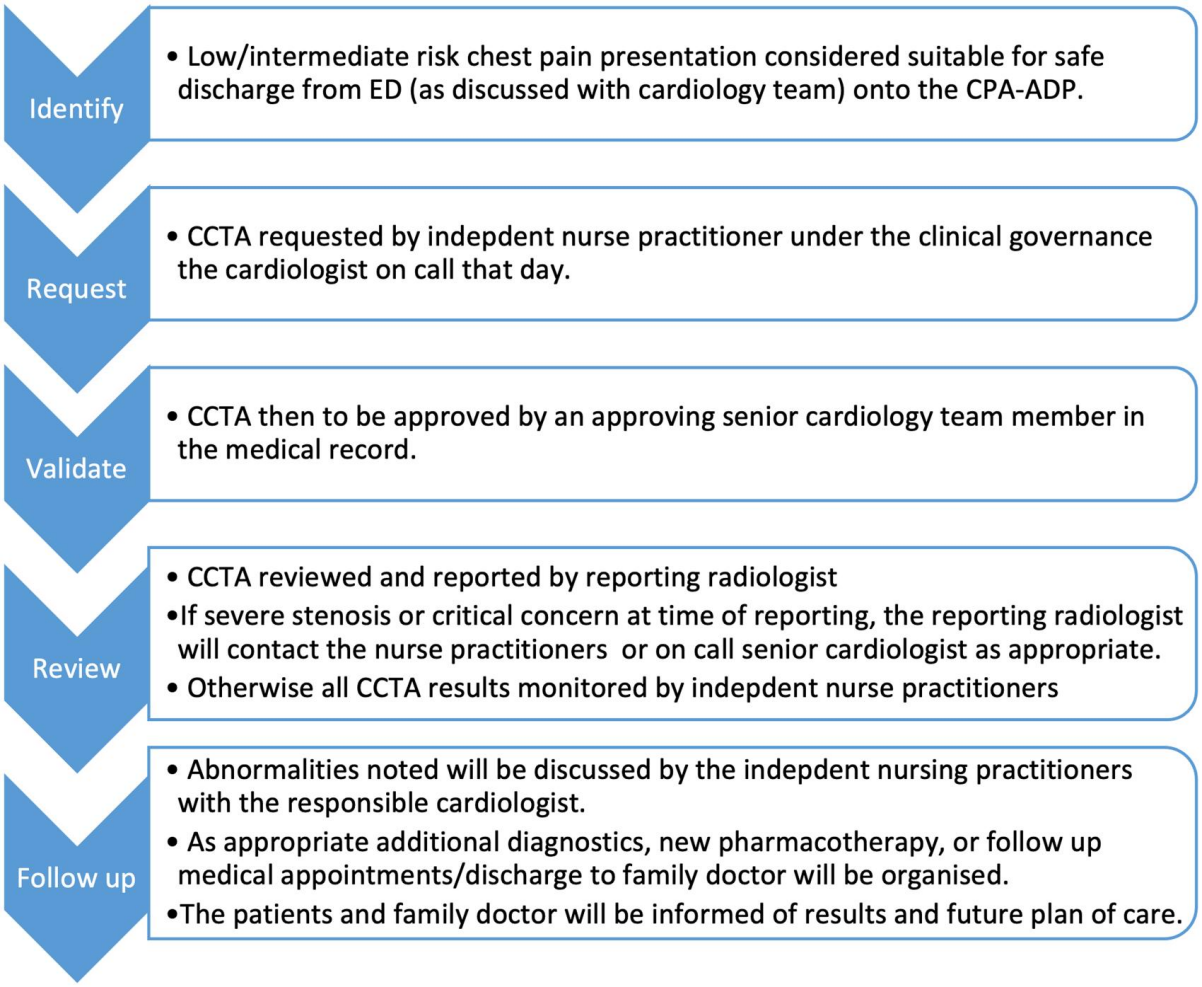
Accelerated diagnostic pathway. This explains the healthcare pathway in a visual format, as the reader can appreciate. This pathway involved different elements of hospital staff: emergency department, cardiology, and radiology. Most patients are followed up in the virtual chest pain clinic; however, in some incidences, findings are clinical significance are escalated at time of reporting. Abbreviations: CCTA = coronary computed tomography angiography; CPA-ADP = chest pain assessment accelerated diagnostic pathway; ED = emergency department.

As in accordance with European guidelines at time of pathway creation,^8^ if the last episode of chest pain is within 6 h of presentation, then the hs-cTn is repeated at 3 h. If these parameters are normal, then the HEART score is calculated, and patients are designated as low risk (0-3) or high risk (>3) in accordance with standard use.^9^ Low-risk patients are either discharged or commence on the CCTA pathway. High-risk patients are referred for further evaluation by cardiology services. After discharge, a virtual chest pain clinic is led by the advanced nurse practitioner and clinical nurse specialist staff at which point the governance transitions to the cardiology department. Figure 1 describes this pathway in a visual format.

An invasive strategy with invasive coronary angiography (ICA) remains the standard of care in non-ST-segment elevation acute coronary syndrome (NSTE-ACS) with high-risk criteria, and in those with a high index of suspicion for unstable angina.^7^ A selective invasive approach is also appropriate in those with NSTE-ACS without high-risk features and a low suspicion for NSTE-ACS.^10^

The ROMICAT-II^11^ and BEACON^12^ trials have described CCTA versus ICA in the management of suspected acute coronary syndrome in low-risk populations (ROMICAT-II no patients with elevated troponin, BEACON 24/500), with favourable results for CCTA in low-risk populations. However, in both of these trials, the CCTA was performed before discharge. CCTA when performed instead of ICA within 72 h of discharge did not experience any major cardiac events at 72 h.^13^ However, there are limited data on patient outcomes related to a delay to CCTA greater than 72 h.

### Aim of study

1. To establish median time to CCTA and associated major adverse cardiac events (MACE) after a presentation to the ED with chest pain.
2. To identify incidence of downstream invasive angiography, coronary revascularization, and use of other healthcare diagnostics.
3. To identify differences in patient characteristics affecting physician choice between CCTA and ICA in low-risk chest pain presentations.

## Methods

### Study design and site

This is a single-centre cohort study at St James Hospital with prospective data collection over January 1, 2023 to December 12, 2023. Patients are grouped into two cohorts (CCTA and ICA) with outcomes of interest recorded (characteristics, results of investigations, abnormal cardiac/non-cardiac findings, and coronary revascularization).

### Inclusion criteria

The inclusion criteria were as follows: haemodynamically stable ambulatory patients over the age of 18 presenting unscheduled to the ED with symptoms suggestive of acute coronary syndrome referred to the early outpatient CCTA pathway, or admitted for invasive angiography.

### Exclusion criteria

The exclusion criteria were as follows: haemodynamically unstable patients, patients with ST elevation myocardial infarction, and patients transferred as inpatients from referring hospitals.

### Definitions

Length of stay for CCTA patients in the ED is expressed in hours. Family history was defined as a history of ischaemic heart disease in a first-degree male relative <55 years, or first-degree female relative <65 years. Cardiac risk factors included both treated and untreated patients. Hypertension was defined as grade 1 arterial hypertension or greater. History of diabetes mellitus was defined as a history of HBA1c >48 mmol/mol, 2 h oral glucose tolerance test ≥11.1 mmol/L, or fasting plasma glucose ≥7mmol/L. Dyslipidaemia included acquired or inherited elevation in total cholesterol, low-density lipoprotein, or triglyceride level. A history of vascular disease included peripheral arterial disease, ischaemic cerebrovascular disease, and cardiovascular disease. These variables included occlusive and non-occlusive disease that may or may not have been revascularized previously. The HEART score is expressed as a median. Systolic and diastolic blood pressures are expressed in mmHg, with heart rate (HR) in beats per minute. ECG is expressed as total number of patients with normal findings. Hs-cTn assay was via Roche Cobas e801 immuoassay analysis and expressed as ng/L. Hs-cTn is expressed as the total number of patients with a normal test result for CCTA patients, and as a median with interquartile range for the ICA cohort. Serum creatinine is expressed as μmol/L. Wait time for CCTA is time from discharge from the ED until CCTA acquisition and is expressed in days. Revascularization includes use of percutaneous coronary intervention and coronary artery bypass surgery and is further defined by inclusion of the proximal left anterior descending artery (LAD) and/or the left main coronary artery (LMCA). Artefact was defined as anything greater than minor level by the reporting radiologist. Dose-length product is expressed in mGy cm.

### Image acquisition

A dual-source CT Siemens Drive with 0.75 mm slice thickness was used for image acquisition. Cardiac scan protocols were based on the patients HR and variability. Patients with a regular HR of under 60 beats per minute underwent prospective ECG-triggered spiral scanning (“Flash”) scan. Patients outside of this underwent retrospective scanning with a focus on either the mid diastolic or systolic window depending on HR.

### Ethical approval

Ethical approval was attained from the St James Hospital/Tallaght University Hospital Joint Research Ethics Committee.

### Statistical analysis

In this study, we leveraged advanced machine learning techniques and Bayesian statistical methods to analyse the data. Specifically, we utilized the Random Forest algorithm, a robust machine learning approach known for its predictive accuracy and ability to handle complex interactions and non-linear relationships. This analysis was conducted in R4.3.1, employing the “randomForestSRC” package, which is specifically designed for survival, regression, and classification random forests.

For the Bayesian logistic regression analysis, we adopted the “rstan” package in R, which facilitates full Bayesian inference using Stan, a state-of-the-art platform for statistical modelling and high-performance statistical computation. This approach allowed us to estimate the odds of independent variables influencing 30-day mortality, incorporating prior knowledge and quantifying uncertainty more effectively than traditional logistic regression models.

The combination of Random Forest for model optimization and Bayesian logistic regression for detailed odds estimation showcases a comprehensive analytical strategy that harnesses the strengths of both machine learning and Bayesian statistics. This integrated approach, utilizing the capabilities of “randomForestSRC” and “rstan” packages in R, provided a robust framework for understanding the factors contributing to 30-day mortality in the study population.

## Results

### Characteristics

Table 1 demonstrates the characteristics of the CCTA population. Please note that continuous results are expressed as a median with interquartile range provided, whereas categorical variables are expressed as a total with a percentage. Median HEART score was 3, suggesting a low-risk population. Majority of patients were male. The median time to outpatient CCTA was 14 days.

**Table 1.** Characteristics of CCTA population.

Total amount Age Hospital stay (days) Gender Family history Hypertension T2DM T1DM Smoker Dyslipidaemia Vascular history HEART score SBP DBP HR ECG normal Troponin normal Creatinine normal Wait time to CCTA Calcium score Dose length product Artefactual scans Angiography Revascularization MI/death at 30 days	369 52 (44-60) 6.5 (5-9) Male = 198 (54), Female = 171 (46) 206 (57) 122 (33) 34 (9) 2 (0.05) Current 96 (26), Ex 92 (25) 163 (44) 7 (2) 3 133 (120-150) 80 (76-88) 76 (68-87) 323 (87.5) 357 (97) 72 (61-85) 14 (5-24) 0 (0-24) 190 (113-276) 35 (9.5) 39 (11) 9 (2)-5 (1) involving LAD/LMCA 0	Normal cardiac findings Luminal stenoses CADRADS Vulnerable plaque Non-luminal findings Non-cardiac findings Statin	219 (59%). 117 (32%) 1 = 58 (15.7), 2 = 26 (7), 3 = 19 (5.2), 4 = 13 (3.5), 5 = 1 (0.2) 13 (4) Intra-atrial septal finding = 8 (2)—PFO = 7 (2), ASD = 1 (0.2). Bridging = 7 (2)—Mid-LAD = 5 (1), distal LAD = 2 (0.4). Valvular calcification = 4 (1). Ascending aortic aneurysms = 3 (0.8). Coronary anomalies = 2 (0.4): LMCA from NCC with no high-risk features. LCx from right sinus. Bicuspid AV = 1 (0.2). Left atrial 1 cm × 1 cm inferior wall appendage = 1 (0.2). Membranous ventricular septal aneurysm = 1 (0.2). Pericardial thickening and fat stranding = 1 (0.2) Normal = 273 (74%). Lung = 53 (14). Nodular changes = 26 (7). Infectious changes = 12 (3). Atelectasis = 7 (2). Emphysema = 3 (0.8). Fibrosis = 3 (0.8). Bronchiectasis = 2 (0.5). Pulmonary trunk dilatation = 1 (0.2). Pleural plaque = 1 (0.2). Dependent changes = 1 (0.2). Pneumonitis = 1 (0.02). Pneumothorax = 1 (0.02). Pleural effusion = 1 (0.02) Stomach = 21 (6). Hiatus hernia = 18 (5). Oesophageal dilatation = 2 (0.5). Gastric band = 1 (0.02) Liver = 13 (4). Cyst = 7 (2). Hepatosteatosis = 5 (1). Haemangioma = 1 (0.2) Breast = 10 (3). Nodules = 5 (1). Gynaecomastia = 3 (0.8). Breast implants = 1 (0.2). Mass = 1 (0.2) Skeletal = 4 (1). Rib fracture = 2 (0.5). Pectus Excavatum = 1 (0.2). Rib sclerosis = 1 (0.2) Vascular = 2 (0.4). Indeterminate vessel coming over pulmonary artery = 1 (0.2). Scattered atheroma in descending thoracic aorta = 1 (0.2) Commenced = 67 (18), Already on = 38 (10), Not prescribed = 11 (3), Dose increased = 5 (1)

Please note that continuous results are expressed as a median with interquartile range provided, whereas categorical variables are expressed as a total with a percentage following. Abbreviations: CCTA = coronary computed tomography angiography; DBP = diastolic blood pressure; ECG = electrocardiogram; HR = heart rate; LAD = left anterior descending artery; LMCA = left main coronary artery; SBP = systolic blood pressure.

### Radiographic findings

The radiographic findings at CCTA are shown in Table 1. Median calcium scoring was 0, suggesting a favourable prognostic outcome. Sublingual glyceryl trinitrate was used in all patients. Institutional protocol first-line rate control strategy was ivabradine per oral (15 mg if resting HR >65 beats per minute, or 7.5 mg if HR 55-65). Most patients had no cardiac (59.3%) or extra-cardiac (73.9%) findings. Abnormal findings are listed by system in the table. For those patients with luminal stenoses, any high-risk plaque features were included by the reporting radiologist in the final summary, with the CAD-RADS classification used for classification.

### Population outcomes and interventions

Table 1 demonstrates outcomes and interventions for the population. Precisely, 19.6% of patients were commenced on high-potency statins (atorvastatin and rosuvastatin) or had their pre-existing statin dosing intensified. Eleven percent of the CCTA patient subsequently underwent ICA, with low level (2%) of overall revascularization, with only 1.4% involving proximal LAD or LMCA. A relatively low amount of patients (5%) required subsequent diagnostics of specialty outpatient referral.

### Factors influencing physician choice between CCTA and ICA

**Figure 2.**
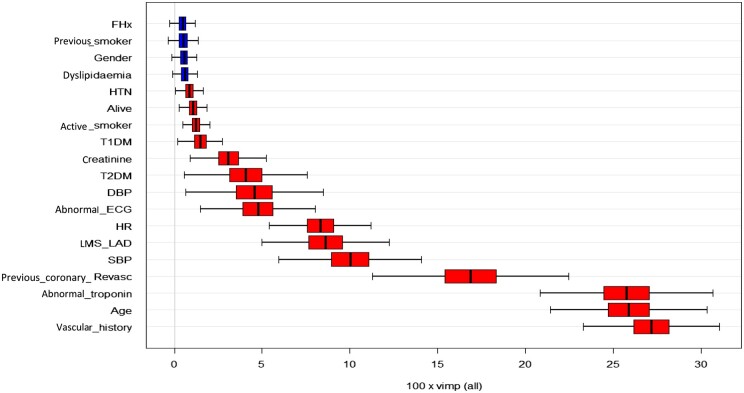
Influence of each variable on physician choice between ICA and CCTA (top). Abbreviations: CCTA = coronary computed tomography angiography; DBP = diastolic blood pressure; ECG = electrocardiogram; HR = heart rate; HTN = hypertension; ICA = invasive coronary angiography; LMS-LAD = involvement of left main steam or left anterior descending; T1DM = type 1 diabetes mellitus; T2DM = type 2 diabetes mellitus.

**Figure 3.**
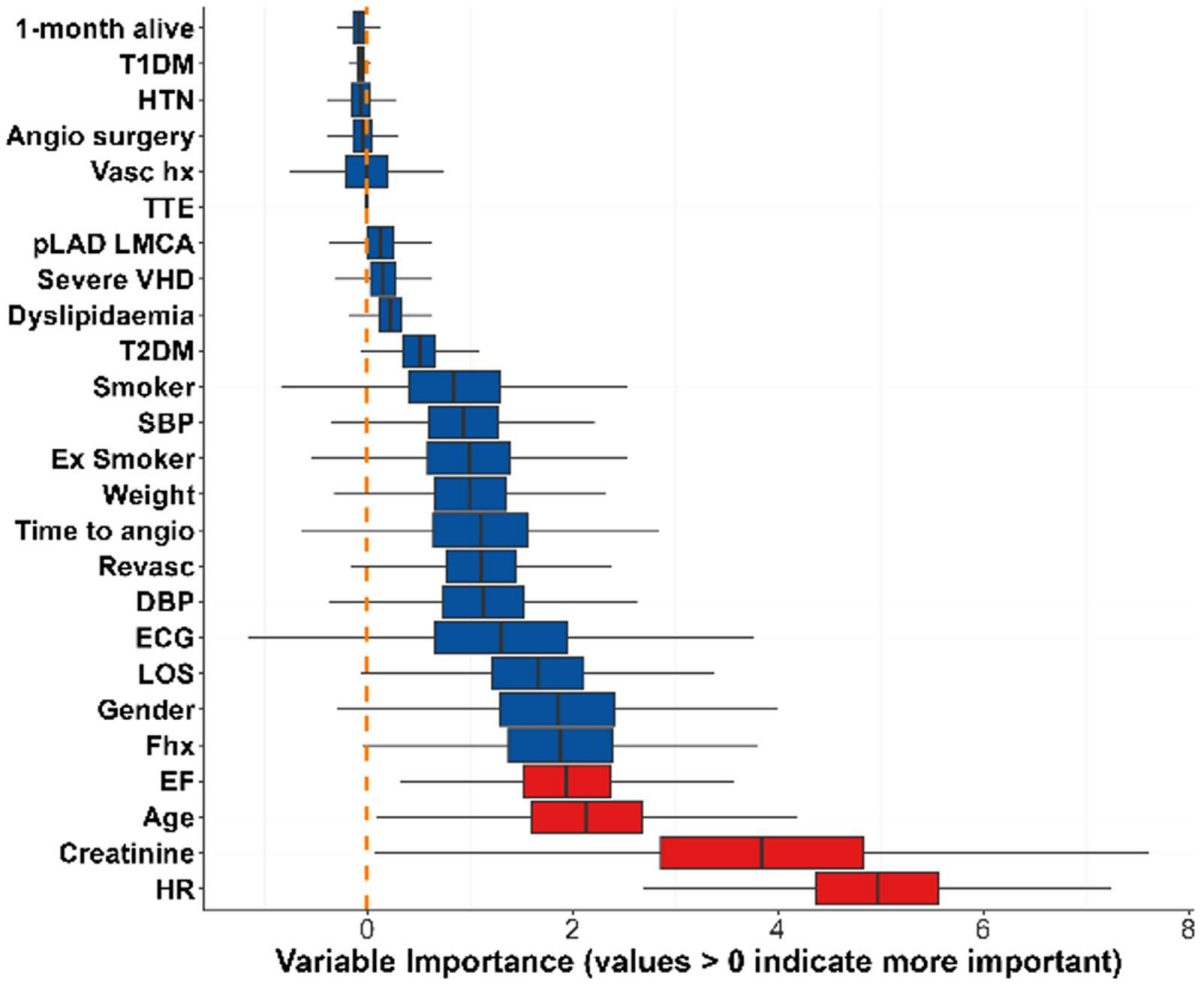
Influence on abnormal troponin level (middle). Abbreviations: DBP = diastolic blood pressure; ECG = electrocardiogram; EF = ejection fraction; HR = heart rate; HTN = hypertension; LMS-LAD = left main coronary artery/left anterior descending artery; LOS = length of stay; pLAD-LMCA = proximal left anterior descending artery or left main coronary artery; SBP = systolic blood pressure; T1DM = type 1 diabetes mellitus; T2DM = type 2 diabetes mellitus; TTE = transthoracic echocardiography; VHD = valvular heart disease.

**Figure 4.**
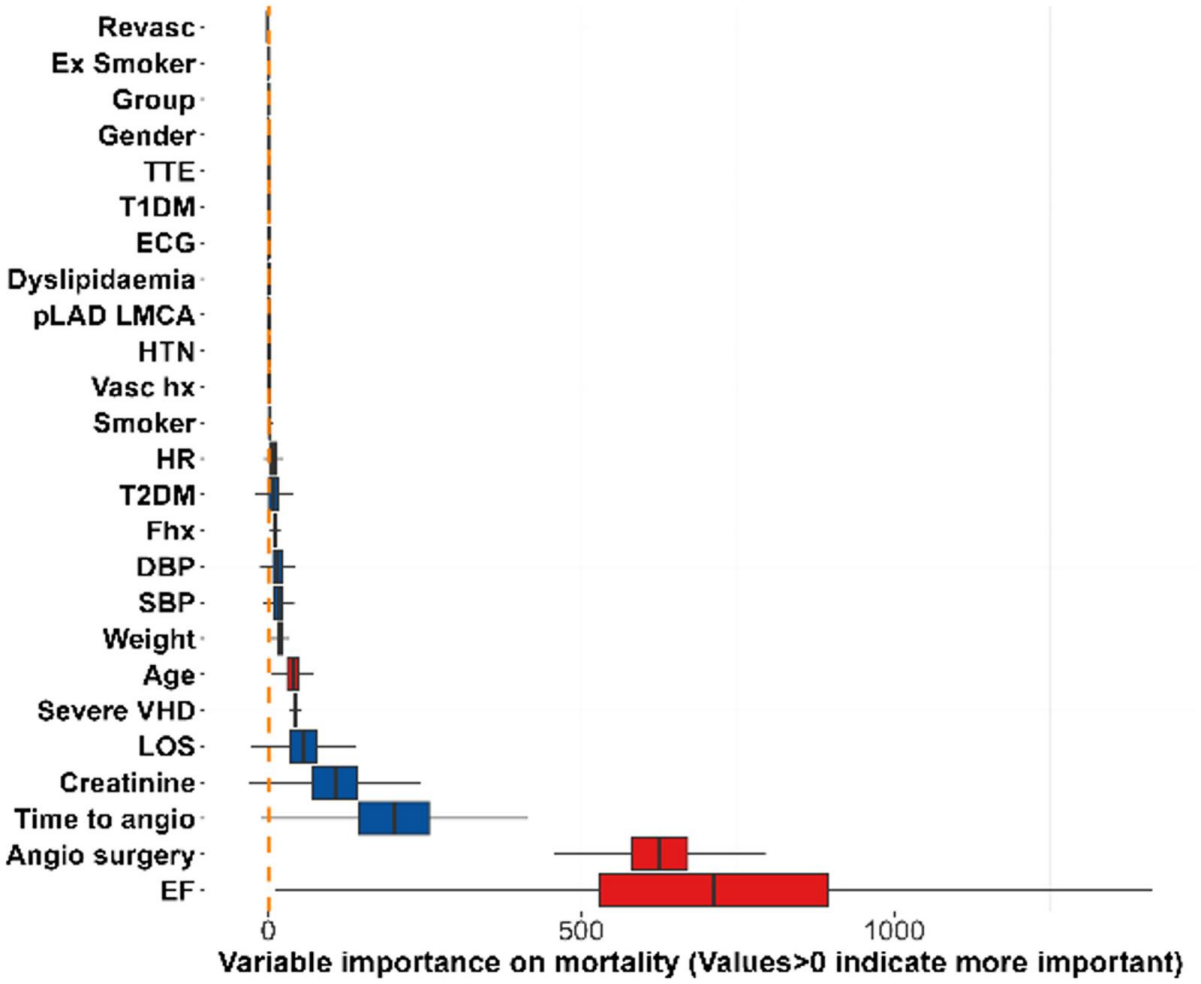
Effect on 1-month mortality (bottom); variables >0 indicate higher importance. Statistical analysis is described in detail in the document—R package was used to test for relationship among variables. Abbreviations: DBP = diastolic blood pressure; ECG = electrocardiogram; EF = ejection fraction; HR = heart rate; HTN = hypertension; LOS = length of stay; pLAD-LMCA = proximal left anterior descending artery or left main coronary artery; SBP = systolic blood pressure; T1DM = type 1 diabetes mellitus; T2DM = type 2 diabetes mellitus; TTE = transthoracic echocardiography; VHD = valvular heart disease.

Regarding choice of test, Figures 2-4 show the influence of each variable on physician choice between inpatient ICA and early outpatient CCTA, influence of each variable regarding an abnormal troponin result, and finally effect on one-month mortality. A history of vascular disease or cardiac revascularization, increased patient age, abnormal troponin, and abnormal systolic blood pressure were the variables of most importance regarding physician choice of imaging.

### Influence of calcium score

According to the results, patients with abnormal calcium score had a higher odd of receiving the treatment (see Table 2). For example, on multivariate analysis, the odds ratio of a composite of statin therapy, performance of angiography, and coronary revascularization was 59.1 for those patients with an abnormal calcium score. It should be noted that 15 patients did not have a calcium score performed.

**Table 2.** Comparison between statin therapy, angiography, and coronary revascularization by calcium status.

Characteristic	Overall, *N* = 369	Normal Ca, *N* = 250	Abnormal Ca, *N* = 119	Odds ratio (95%CI)	*P* value
Composite of statin therapy, angiography, and revascularization	283 (77%)	241 (97%)	42 (35%)	59.1	<0.001
	85 (23%)	8 (3.2%)	77 (65%)		
Underwent angiogram	330 (89%)	246 (98%)	84 (71%)	29.59	<0.001
	39 (11%)	4 (1.6%)	35 (29%)		
Statin therapy commenced	297 (80%)	242 (97%)	55 (46%)	29.2	<0.001
	72 (20%)	8 (3.2%)	64 (54%)		
Coronary revascularization	360 (98%)	249 (100%)	111 (93%)	37.4	<0.001
	9 (2.4%)	1 (0.4%)	8 (6.7%)		

### Population characteristic differences between CCTA and ICA

Tables S1 and S2 demonstrate the differences between the CCTA and stable ICA population, and differences between ICA population when defined by normal/abnormal troponin. The CCTA population were a younger population with a higher proportion of females. Abnormal troponin was associated with older patients, with a higher length of hospital stay in those undergoing ICA. Admission troponin had a significant relationship with admission HR and serum creatinine, ejection fraction, revascularization, and 30-day mortality. The indications for angiography in the troponin <14 cohort varied: unstable angina (56), stable angina (24), atypical chest pain (22), accelerated angina (7), atrioventricular block/sinus node dysfunction (6), heart failure reduced ejection fraction (6), symptomatic severe aortic stenosis (4), atrial tachyarrhythmias (4), and severe mitral regurgitation, decompensated heart failure, symptomatic hypertension, and pulmonary hypertension work up (all 1).

## Discussion

### Patient safety

This study demonstrates similar good safety endpoints when compared to previous comparable literature.^13^ This is in the context of a median time to outpatient cardiac CT for low-risk chest pain of 14 days, where in our medium-sized population, no patient suffered MACE at 30 days. Existing literature refers to earlier testing at under 72 h,^13^ and it is notable that our median time far beyond this in the semi-acute setting. The median time to CCTA is due to availability of resources rather than by design. However, it is known that low-risk chest pain patients have a very low risk of short- and long-term MACE, and expedition of early non-invasive testing has not been shown to benefit patients.^14^ Admission of patients for early non-invasive testing has a minor effect on outcomes, however, with a large number needed to treat: 500 to avoid 1 MI, 333 to avoid 1 death, and 200 to avoid MACE at 30 days.^15^

### Pharmacological patient interventions

One of the strengths of CCTA lies in the risk stratification and pharmacological management of non-occlusive coronary disease with a reduction in (non)fatal myocardial infarction.^1^^,^^16^ One-fifth of our patients had non-occlusive coronary artery disease identified and managed via pharmacotherapy in the virtual chest pain clinic, with 32% found to have combined obstructive and non-obstructive coronary stenoses. This is only half than that stated in the SCOT-HEART trial^1^ of 63%, and less than Scheuermeyer et al^13^ (45%), although our incidence of a normal calcium score is slightly higher (68% vs. 65%) than SCOT-HEART. Our population markedly differed in the incidence of CAD-RADS stenoses of 4/5 when compared to SCOT-HEART (4% vs. 25%) but was more comparable to Scheuermeyer (4% vs. 3%). We also had a lower incidence of CAD-RADS 3 and below stenoses (28% vs. 38% in SCOT-HEART and 41% by Scheuermeyer).

The rate of commencement of preventative therapy is very similar to SCOT-HEART (19.4%). Despite the differences in the populations risk factor profile, this is probably accounted for as much more patients at baseline in SCOT-HEART were on prescribed statin therapy (10% vs. 44%). Our CCTA cohort is younger (52 vs. 57 years), with less incidence of cardiovascular risk factors than the SCOT HEART population: combined smoking, 51% vs. 53%; arterial hypertension, 33% vs. 35%; diabetes mellitus, 10% vs. 11%; dyslipidaemia, 44% vs. 53%. This suggests our cohort is among the lower risk of published cohorts. Ultimately, we feel that the younger age, less cardiovascular risk factors at baseline, and higher prevalence of normal calcium score accounts for the differences between our population and that of SCOT-HEART.

### Invasive and other downstream patient interventions

Despite only having half the incidence of coronary artery disease on CCTA, our rate of invasive angiography is only slightly lower than SCOT-HEART but still comparable (11% vs. 13% in CCTA and standard care arm). Ten percent in SCOT-HEART underwent functional assessment after CT, as opposed to 1% in our population. This reflects a local access issue, where frequently it is more timely to pursue ICA with invasive functional assessment than sequential non-invasive functional assessment. Table S3 demonstrates the other downstream investigations performed.

Incidental findings were comparable to other studies.^17^ Non-luminal cardiac anomalies (7% vs. 7%), as well as gastrointestinal (9% vs. 9%), and lung (14% vs. 14%) remained markedly similar.

### Influence of calcium score

Calcium score heavily influences physician behaviour in this study—the odds ratio of statin therapy (29.2), performance of angiography (29.6), revascularization (37.4), and the composite of all three (59.1) reflects this. Previous data have shown that a high calcium score can influence physician behaviour in this regard.^18^ Our data are novel in that this extends across all abnormal calcium scores.

### CCTA and ICA

Patients who underwent CCTA were different from those for ICA in our study. The CCTA population were a younger population with a higher proportion of females. A history of vascular disease or cardiac revascularization, increased patient age, abnormal troponin, and abnormal systolic blood pressure were the variables that were found to have a notable correlation with physician choice of imaging. These differences in patient characteristics were anticipated by both the pathway and study design, as lower risk patients (by risk factors and basic investigations) are anticipated to proceed to the CCTA pathway.

### Artefact

Our study quotes a high number of clinically relevant artefacts at reporting (9.5%) when compared to other published data.^19^ Real-world data are lacking in the literature, including landmark trials.^1^^,^^20^^,^^21^ Consensus on an objective assessment of artefact at time of reporting should be considered, like as in the CAD-RADS scoring method. It is important to note that there could be a high level of inter-observer variability when it comes to this topic.

### Translation to other jurisdictions

The Republic of Ireland is considered a moderate risk for cardiovascular mortality by the World Health Organization.^22^ Countries in a similar risk category include Germany, Italy, Portugal, Iceland, Sweden, Finland, Greece, Austria, and Slovenia. It is not unreasonable to expect some level of similarity in patient trends in these countries. Our high prevalence of a normal calcium score in this type of population is roughly similar to published meta-analyses^23^ (67% vs. 60%), again pointing to the fact that our population retains a low-risk profile.

This study demonstrates a real-world review of a medium-sized population in a public university hospital that is comparable to many other health systems with a strain on public resources. It shows that in this cohort, there was no mortality or MI when CCTA was performed at a median of several weeks after discharge from ED. This study encourages other countries with a similar cardiovascular risk profile to invest in an accelerated diagnostic pathway with CCTA for low-risk chest patients.

## Limitations

The findings of this study are limited as a single-centre observational study. The comparators between the CCTA and ICA population are susceptible to confounding bias despite best efforts to account for this in the statistical methods. The results of this study cannot be used to suggest that patients who undergo CCTA vs. ICA have a lower rate of mortality, nor that CCTA reduces the likelihood of coronary revascularization vs. ICA. The lower rates of coronary artery disease observed in comparison to the described comparator studies may reflect an eagerness to utilize a newly approved patient pathway with very low-risk patients.

## Clinical perspective

Competency in patient care: The low-risk patient undergoing outpatient cardiac CT at two weeks after an ED presentation can be reassured that the likelihood of a major adverse cardiac event at 30 days is extremely low.

Translational Outlook 1: A consensus of objective quantification of artefact in CCTA at time of reporting.

Translational Outlook 2: The publication of data regarding other accelerated diagnostic pathways in separate jurisdictions.

## Conclusion

A median of 14 days to CCTA for low-risk chest pain presentations to the ED resulted in no mortality or myocardial infarction at 30 days, with a low level of overall coronary revascularization. Cardiovascular risk factors and biochemical findings appear to influence a cardiologist choice between referral for CCTA and ICA. An abnormal calcium score affected physician behaviour regarding statin therapy, performance of coronary angiography, and coronary revascularization.

## Supplementary Material

tqae119_Supplementary_Data

## Data Availability

All data are available upon request.
