# Supplementary material for: The early after discharge cardiac CT for low-risk chest pain study: the ED-CT study
Source: Br J Radiol. 2024 Jun 18;97(1160):1483–91. doi: 10.1093/bjr/tqae119 (PMC11256939; doi:10.1093/bjr/tqae119)
Supplement: tqae119_Supplementary_Data [file tqae119_supplementary_data.zip › tqae119_Supplementary_Data/Table S1.docx]

Table 3: Characteristics of CCTA population and ICA population

|  | **CCTA (N = 369)** | **ICA (N = 375)** |  |
| --- | --- | --- | --- |
| **Age** | 52 (44-59.5) | 65 (59 – 73) | **<0.001** |
| **Gender** | Male = 198 (53.7%)  Female = 171 (46.3%) | Male = 262 (69.9%)  Female = 113 (30.1%) | **<0.001** |
| **Family history** | 206 (56.94%) | 121 (32.3%) | **<0.001** |
| **Hypertension** | 122 (33%) | 228 (60.8%) | **<0.001** |
| **T2DM** | 34 (9.2%) | 87 (26.1%) | **<0.001** |
| **T1DM** | 2 (0.05%) | 3 (0.8%) | >0.99 |
| **Smoker** | Current = 96 (26%)  Ex = 92 (24.9%) | Current = 90 (24%)  Ex = 150 (40%) | 0.512  **<0.001** |
| **Dyslipidaemia** | 163 (44.2%) | 230 (61.3%) | **<0.001** |
| **Vascular history** | Total = 10 (2.7%)  PVD 3 (0.8%)  CVD 3 (0.8%)  IHD 1 (0.2%) | Total = 158 (42.1%)  IHD = 37 (9.9%)  PVD = 15 (4%)  PCI = 49 (13.1%)  CABG = 25 (6.7%)  CABG and PCI = 6 (1.6%)  CVD = 19 (5.1%)  PCI and CVD = 2 (0.5%)  PVD and PCI = 1 (0.3%)  IHD and PVD and CVD = 1 (0.3%)  IHD and PVD = 2 (0.5%)  IHD and CVD = 1 (0.03%) | **<0.001** |
| **SBP** | 133 (120-150) | 137 (121-154) | 0.195 |
| **DBP** | 80 (76-88) | 78 (72 – 87) | **0.003** |
| **HR** | 76 (68-87) | 78 (68 – 90) | 0.075 |
| **ECG** | Normal = 323 (87.5%) | Normal = 253 (67.5%) | **<0.001** |
| **Troponin** | Normal = 357 (96.7%) | 20 (1 – 62) | **<0.001** |
| **Creatinine** | 72 (61-85) | 81 (68 – 99) | **<0.001** |
| **Revascularization** | 9 (2.4%)   - PCI 6 - CABG 3   Pending angiography = 8 | 164 (43.7%)   - PCI = 135 (36%) - CABG = 27 (7.2%) | **<0.001** |
| **pLAD/LMCA involvement** | 5 (1.4%) | 85 (22.7%) | **<0.001** |
| **MI at 30 days** | 0 | 0 |  |
| **Death at 30 days** | 0 | 9 (2.4%) | **0.004** |

Addendum: 1 patient family history unknown
