# Supplementary material for: The early after discharge cardiac CT for low-risk chest pain study: the ED-CT study
Source: Br J Radiol. 2024 Jun 18;97(1160):1483–91. doi: 10.1093/bjr/tqae119 (PMC11256939; doi:10.1093/bjr/tqae119)
Supplement: tqae119_Supplementary_Data [file tqae119_supplementary_data.zip › tqae119_Supplementary_Data/Table S2.docx]

Table 4: Variables in ICA population when separated by initial troponin result

|  | **Troponin <14 (n = 133)** | **Troponin >14 (n = 225)** |  |
| --- | --- | --- | --- |
| **Age** | 63 (55 – 69.5) | 68 (61 – 76) | **<0.001** |
| **Gender** | Male = 83 (62.4%)  Female = 50 (37.6%) | Male = 164 (72.9%)  Female = 61 (27.1%) | **0.038** |
| **LOS (days)** | 4.1 (2.38 – 8.19) | 7 (3.1 – 16.06) | **<0.001** |
| **Time to angiography (days)** | 2.15 (1.27 - 4) | 2.08 (1.08 – 5.23) | 0.936 |
| **Family history** | 63 (47.4%) | 57 (25.3%) | **<0.001** |
| **HTN** | 80 (60.2%) | 137 (60.1%) | 0.89 |
| **T2DM** | 30 (22.6%) | 52 (23.1%) | 0.904 |
| **T1DM** | 0 | 3 (1.3%) | 0.298 |
| **Smoker** | Current = 35 (26.3%)  Ex = 47 (35.3%) | Current = 49 (21.8%)  Ex = 97 (43.1%) | 0.379  0.147 |
| **Dyslipidaemia** | 91 (68.4%) | 130 (57.8%) | 0.079 |
| **PVD/CVD/IHD** | Total = 56 (42.1%)  CABG = 5 (4%)  PCI = 18 (14.3%)  IHD = 19 (5.1%)  CABG and PCI = 2 (0.5%)  CVD = 8 (2%)  PCI and CVD = 1 (0.8%)  PVD and PCI = 1 (0.8%)  IHD and PVD and CVD = 1 (0.8%)  CABG and CVD = 1 | Total = 96 (42.6%)  CABG = 18 (8.6%)  PCI = 29 (13.1%)  IHD = 15 (6.6%)  PVD = 14 (6.1%)  CABG and PCI = 4 (1.9%)  CVD = 12 (5.6%)  PCI and CVD = 1 (0.5%)  PVD and IHD = 1 (0.5%)  IHD and CVD = 2 (1%) | 0.079 |
| **SBP** | 140 (125 – 158) | 135 (121 – 154) | 0.382 |
| **DBP** | 79 (72 – 87) | 78 (71 – 88) | 0.901 |
| **HR** | 73 (66 – 84) | 81 (70 – 95) | **<0.001** |
| **Weight** | 82.6 (70.9 – 89.9) | 79 (68.25 – 90.75) | 0.315 |
| **ECG** | Normal = 99 (78.6%) | Normal = 134 (59.6%) | **<0.001** |
| **Troponin** | <14 = 133 | 45 (23 – 115) |  |
| **Creatinine** | 73 (64 – 85) | 88 (71 – 106) | **<0.001** |
| **TTE** | N = 99 (74.4%) | N = 201 (89.3%) | **<0.001** |
| **EF** | 53 (47 – 57) | 50 (36 – 55) | **<0.001** |
| **Severe VHD** | Total = 5 (3.8%)  AS = 4 (3%)  MR = 1 (0.8%) | Total = 24 (10.7%)  AS = 7 (2.7%)  MR = 7 (2.7%)  MR and TR = 4 (1.8%)  AR and MR = 2 (1%)  AS and MR = 1 (0.5%)  AS and MS = 1 (0.5%)  AS and TR = 1 (0.5%)  TR = 1 (0.5%) | **0.021** |
| **Revascularization** | 45 (33.8%)   - PCI = 38 (28.6%) - CABG = 7 (5.2%) | 114 (50.7%)   - PCI 95 (42.2%) - CABG 18 (8%) - POBA 1 (0.5%) | **0.003** |
| **pLAD/LMCA involvement** | 25 (18.8%) | 58 (25.8%) | 0.109 |
| **Angiogram as part of surgical work up** | Total = 4 (3%)  SAVR = 1 (0.8%)  TAVI = 2 (1.5%)  MVR = 1 (0.8%) | Total = 19 (8.4%)  TAVI = 8 (3.5%)  MVR = 4 (1.8%)  MVR/TVR = 3 (1.3%)  AVR/MVR/root replacement = 1 (0.5%)  AVR/MVR = 1 (0.5%)  SAVR = 1 (0.5%)  Left SFA bypass = 1 (0.5%) | **0.043** |
| **Death at 30 days** | 0 | 8 (3.5%) | **0.028** |
| **MI at 30 days** | 0 | 0 |  |

Addendum: Weight not done in 10 patients with normal troponin, not done in 8 patients with abnormal troponin. Troponin level not performed in one death at 30 days. 17 patients from the overall cohort did not have a troponin performed during their admission and so are omitted from this table.
