# Supplementary material for: The early after discharge cardiac CT for low-risk chest pain study: the ED-CT study
Source: Br J Radiol. 2024 Jun 18;97(1160):1483–91. doi: 10.1093/bjr/tqae119 (PMC11256939; doi:10.1093/bjr/tqae119)
Supplement: tqae119_Supplementary_Data [file tqae119_supplementary_data.zip › tqae119_Supplementary_Data/Table S3.docx]

Table 5: Subsequent referrals and performed radiology

| **Subsequent radiology/referrals** | Total = 13 (4%)  Cardiac MRI = 3  TTE = 2  CT thorax = 1  US breast = 1  TOE = 1  OGD = 1  Repeat CCTA = 1  MRI thorax = 1  EST = 1  Repeat CXR = 1 | Total = 4 (1%)  Rapid Access Lung OPD = 2  Haematology referral = 1  Breast OPD referral = 1 |
| --- | --- | --- |
